# MicroRNA-21 Plays Multiple Oncometabolic Roles in Colitis-Associated Carcinoma and Colorectal Cancer via the PI3K/AKT, STAT3, and PDCD4/TNF-α Signaling Pathways in Zebrafish

**DOI:** 10.3390/cancers13215565

**Published:** 2021-11-06

**Authors:** Chi-Yu Lai, Kun-Yun Yeh, Bi-Feng Liu, Tzu-Ming Chang, Chuan-Hsun Chang, Yung-Feng Liao, Yi-Wen Liu, Guor Mour Her

**Affiliations:** 1 Institute of Biopharmaceutical Sciences, National Yang Ming Chiao Tung University, Taipei 112, Taiwan; c.y.stephen.lai@gmail.com (C.-Y.L.); vicky020185@gmail.com (B.-F.L.); a0975108131@gmail.com (Y.-W.L.); 2 Division of Hemato-Oncology, Department of Internal Medicine, Chang-Chung Memorial Hospital, Keelung 204, Taiwan; yehtyng@gmail.com; 3 Division of Surgical Oncology, Department of Surgery, Cheng Hsin General Hospital, Taipei 112, Taiwan; Tzumchang@hotmail.com (T.-M.C.); hsunfang@gmail.com (C.-H.C.); 4 Division of General Surgery, Cheng Hsin General Hospital, Taipei 112, Taiwan; 5 Laboratory of Molecular Neurobiology, Institute of Cellular and Organismic Biology, Academia Sinica, ICOB 238, 128 Sec. 2 Academia Rd., Taipei 11529, Taiwan; yliao@gate.sinica.edu.tw

**Keywords:** microRNAs, colorectal cancer (CRC), inflammatory bowel disease (IBD), colitis-associated colorectal cancer (CAC), zebrafish

## Abstract

**Simple Summary:**

The PI3K/AKT, STAT3, and PDCD4/TNF-α signaling networks, regulated by the microRNA (miR)-21, are critical for inflammatory regulation, tumor suppressor modulation, and oncogenic activation. We developed a zebrafish model (ImiR-21) with an inducible overexpression of miR-21, specifically in the intestine. The miR-21 overexpression resulted in the development of colorectal cancer (CRC) due to inflammatory bowel disease. Furthermore, the physiological, metabolic, and histological aspects of CRC were similar to those of colitis-associated cancer (CAC) induced by the intestinal carcinogens azoxymethane or dextran sodium sulfate in this model. Thus, miR-21 is critical to the pathogenesis of CRC/CAC and could serve as a novel therapeutic target to treat CRC/CAC.

**Abstract:**

Colorectal cancer (CRC) is a leading cause of cancer-related mortality worldwide. Patients with inflammatory bowel disease (IBD) have a high risk of developing CRC. Inflammatory cytokines are regulated by complex gene networks and regulatory RNAs, especially microRNAs. MicroRNA-21 (miR-21) is amongst the most frequently upregulated microRNAs in inflammatory responses and cancer development. miR-21 has become a target for genetic and pharmacological regulation in various diseases. However, the association between inflammation and tumorigenesis in the gut is largely unknown. Hence, in this study, we generated a zebrafish model (ImiR-21) with inducible overexpression of miR-21 in the intestine. The results demonstrate that miR-21 can induce CRC or colitis-associated cancer (CAC) in ImiR-21 through the PI3K/AKT, PDCD4/TNF-α, and IL-6/STAT3 signaling network. miR-21 activated the PI3K/AKT and NF-κB signaling pathways, leading to initial inflammation; thereafter, miR-21 and TNF-α repressed PDCD4 and its tumor suppression activity. Eventually, active STAT3 stimulated a strong inflammatory response and activated the invasion/metastasis process of tumor cells. Hence, our findings indicate that miR-21 is critical for the development of CRC/CAC via the PI3K/AKT, STAT3, and PDCD4/TNF-α signaling networks.

## 1. Introduction

Colorectal cancer (CRC), also known as colon, bowel, or rectal cancer, is a type of cancer that develops in the colon or rectum. In 2020, CRC was the second most common cancer and the second leading cause of mortality due to cancer worldwide [1]. Most CRCs develop from polyps on the inner lining of the colon or rectum. Not all polyps become malignant, but some can transform into cancer over time. Adenomatous polyps (adenoma, a precancerous condition) are an example of polyps that can transform into cancer. Hyperplastic and inflammatory polyps are more common than adenomas but are generally not precancerous. Sessile serrated polyps (SSPs) and traditional serrated adenomas (TSAs) have a higher risk of tumorigenesis of CRC [2]. Although CRC can occur at any age, it is more common in older adults. Moreover, people with a family history of CRC, adenomatous polyps, and inflammatory bowel disease (IBD) are at a high risk of developing CRC.

Familial adenomatous polyposis (FAP), hereditary nonpolyposis colorectal cancer syndrome (HNPCC), and IBD are the top three risk factors for CRC [3]. Crohn’s disease (CD) and ulcerative colitis (UC) are two major forms of IBD [4]. All the abovementioned cancers are categorized as colitis-associated colorectal cancers (CACs). Previous studies have shown that chronic intestinal inflammatory conditions in the gut promote the development of CAC [5,6,7]. Inflammatory or immune cells produce cytokines that affect cell proliferation and apoptosis, promoting tumorigenesis [8]. Proinflammatory cytokines, such as interleukin (IL)-6, IL-11, IL-17, and tumor necrosis factor (TNF)-α, cause GI cancers [9,10,11,12,13]. IL-6, mainly produced by myeloid cells, is a key regulator of tumorigenesis [14]. IL-6 activates the downstream signal transducer and activator of transcription-3 (STAT3) to promote cell proliferation [15,16]. Evidence indicates the role of STAT3 in the development of IBD-related CRC [17,18]. The IL-6/STAT3 signaling pathway regulates tumor cell proliferation [19]. Programmed cell death 4 (*PDCD4*), a celebrated tumor-suppressive gene, is also associated with CRC [20,21]. PDCD4 is well known for its ability to inhibit tumor growth, metastasis, and invasion [22]. In addition, PDCD4 has been shown to be associated with inflammatory diseases [23,24,25].

Clinical evidence demonstrated that microRNA (miRNA)-21 (miR-21) expression was significantly upregulated in the colon tissue of both CD and UC patients compared to that of non-IBD controls [26,27]. miR-21 promotes invasion, intravasation, and metastasis in CRC by downregulating PDCD4 [28]. Furthermore, IL-6/STAT3 signaling stimulates inflammatory pathways in UC by activating the expression of miR-21 [26]. Moreover, PDCD4 deficiency in mouse models aggravates colitis and colitis-associated CRC by promoting the IL-6/STAT3 pathway [20]. However, the molecular mechanisms mediated by miR-21 in IBD and CRC are unclear.

Zebrafish are a remarkable vertebrate model organism for biological and medical studies [29]. Over 70% of human genes have orthologs in zebrafish [30]. Compared to human cell lines or genetically engineered mouse models, zebrafish provide unique insights into the progression and pathogenesis of cancer. The features of cancer development in zebrafish are similar to those observed in humans [31,32]. Hence, in recent years, zebrafish are being increasingly used as model organisms for cancer research [33,34,35]. The cellular composition, architectural organization, and the digestive and immune functions of the intestinal tract is conserved between zebrafish and mammals [36]. However, studies examining the transgenic zebrafish as a model for GI cancer research are scarce [37,38,39].

Studies on various cancer cell lines and animal models indicate that miR-21 promotes colorectal oncogenesis and hence is referred to as an oncomiR [40,41]. In our recent study, miR-21 played multiple oncometabolic roles in nonalcoholic steatohepatitis-associated hepatocellular carcinoma (NAHCC) [42]. Hence, in this study, we aim to examine the time profiles of expression of miR-21 and its target genes during CRC genesis using an oncomiR-21 transgenic zebrafish model (ImiR-21) developed in our laboratory.

## 2. Materials and Methods

### 2.1. Ethics Statement

All animals were maintained in compliance with the Institutional Animal Care and Use Committee (IACUC) guidelines.

### 2.2. Zebrafish Lines and Maintenance

The transgenic zebrafish line ImiR-21 [*Tg(fabp2a:Tet^on^-2A-ZsGreen*, *TRE:mCherry-miR-21*] showed inducible and intestine-specific expression of *mCherry* (red fluorescent protein). Production of miR-21 was driven by the zebrafish *fabp2* promoter. The fish were maintained in a controlled environment with a 14/10-h light-dark cycle at 28 °C.

### 2.3. Doxycycline (Dox) Treatment

The zebrafish embryos and juvenile adults were treated with Dox at a concentration of 25 μg/mL (Sigma-Aldrich, St. Louis, MO, USA) in six-well plates and 3 L tanks, respectively, and the water was changed daily.

### 2.4. Chemical Treatment

To screen for inflammatory markers in the intestine of zebrafish, dextran sodium sulfate (DSS, Sigma-Aldrich) was used to induce colitis in zebrafish. DSS (10 ng/4.6 nL) was injected into the zebrafish duct of Cuvier at 14 and 16 days post-fertilization (dpf). Azoxymethane (AOM, 100 ng/4.6 nL, Sigma-Aldrich) was injected into the duct of Cuvier at 14, 16, and 18 dpf.

### 2.5. RNA Analysis

Total RNA was extracted from intestinal tissue using TRIzol Reagent (Thermo Fisher Scientific, Waltham, MA, USA). cDNA was synthesized using the RevertAid RT Kit (Thermo Fisher Scientific). Real-time quantitative reverse transcription PCR (RT-qPCR) analysis using Fast SYBR Green Master Mix (Thermo Fisher Scientific) was performed on a StepOne Real-Time PCR System (Thermo Fisher Scientific). Expression data were normalized to those of *gapdh* or *U6* of zebrafish. The genes and their corresponding primer sequences are listed in Table S1.

### 2.6. Whole-Mount Alcian Blue Staining

The 4% PFA (paraformaldehyde)-PBS fixed embryos were immersed in acid ethanol (1% HCl in 70% ethanol) for 5 min at room temperature. The embryos were then transferred into alcian blue solution (0.1% alcian blue (Sigma-Aldrich), 20% acetic acid, 80% ethanol) for 16 h at 4 °C with gentle shaking. The alcian blue solution was then replaced with ethanol. Stained embryos were stored in the dark at 4 °C.

### 2.7. Histopathology

Freshly dissected tissues were rinsed with PBS and fixed with 1× Zinc Formal-Fixx (Thermo Fisher Scientific). Fixed tissues were then dehydrated with ethanol and embedded in paraffin (Surgipath Paraplast Plus, Leica Biosystems Division of Leica, Buffalo Grove, IL, USA). Blocks were sliced into 4 μm sections and stained with H&E (hematoxylin and eosin) and alcian blue solution (pH 2.5).

### 2.8. Western Blotting Analysis

Total intestinal proteins were isolated using 1× RIPA buffer (#9806, Cell Signaling Technology, Danvers, MA, USA). Protein concentration was determined using a protein assay kit (500-0001, Bio-Rad, Hercules, California, USA). Protein samples (10–25 μg) were separated using 12% SDS-PAGE and transferred onto PVDF membranes. After blocking with 5% non-fat dried milk in PBS-0.1% Tween 20 (PBST; pH 7.4), membranes were incubated overnight at 4 °C with the following antibodies: anti-PTEN (1:1000, 138G6, Cell Signaling Technology), anti-phosphorylated (p)-AKT (1:2000, Cell Signaling D9E), anti-p-Stat3 (1:1000, D128-3, MBL International, Woburn, MA, USA), anti-PDCD4 (1:1000, sc-376430, Santa Cruz Biotechnology, Santa Cruz, CA, USA), and anti-GAPDH (1:10,000, GTX100118, GeneTex, Hsinchu City, Taiwan, R.O.C.). Protein levels were detected using horseradish peroxidase-conjugated anti-mouse and anti-rabbit IgG (1:5000, AB_10015289 and AB_2313567 Jackson Immuno Research, West Grove, PA, USA) and peroxidase-catalyzed chemiluminescence (WBKLS0100, Merck Millipore, St. Louis, MO, USA).

### 2.9. Immunohistochemistry

The 4 μm tissue sections were incubated overnight at 4 °C with the following antibodies: anti-Pten (1:500, 138G6, Cell Signaling Technology), anti-phospho-AKT (1:200, D9E, Cell Signaling Technology), anti-phospho-Stat3 (1:200, D128-3, MBL International), and anti-PDCD4 (1:500, sc-376430, Santa Cruz Biotechnology). Biotin-conjugated anti-mouse and anti-rabbit IgG (1:500, #31800 and #1820, Thermo Fisher Scientific) were used as secondary antibodies. The VECTASTAIN ABC Kit (PK-6100, Vector Laboratories, Burlingame, CA, USA) was used for the detection. All sections were counterstained with hematoxylin.

### 2.10. Statistical Analysis

All data are presented as mean ± standard error of the mean (SEM). Kaplan–Meier with log-rank test (build in GraphPad Prism 8.0) was used for survival analysis. The number of zebrafish larvae for survival analysis are listed in Table S2. All analyses were performed using GraphPad Prism 8.0 software (GraphPad, San Diego, CA, USA). Differences were considered statistically significant at *p* < 0.05.

## 3. Results

### 3.1. Generation of the Transgenic Zebrafish Line ImiR-21

**Figure 1:**
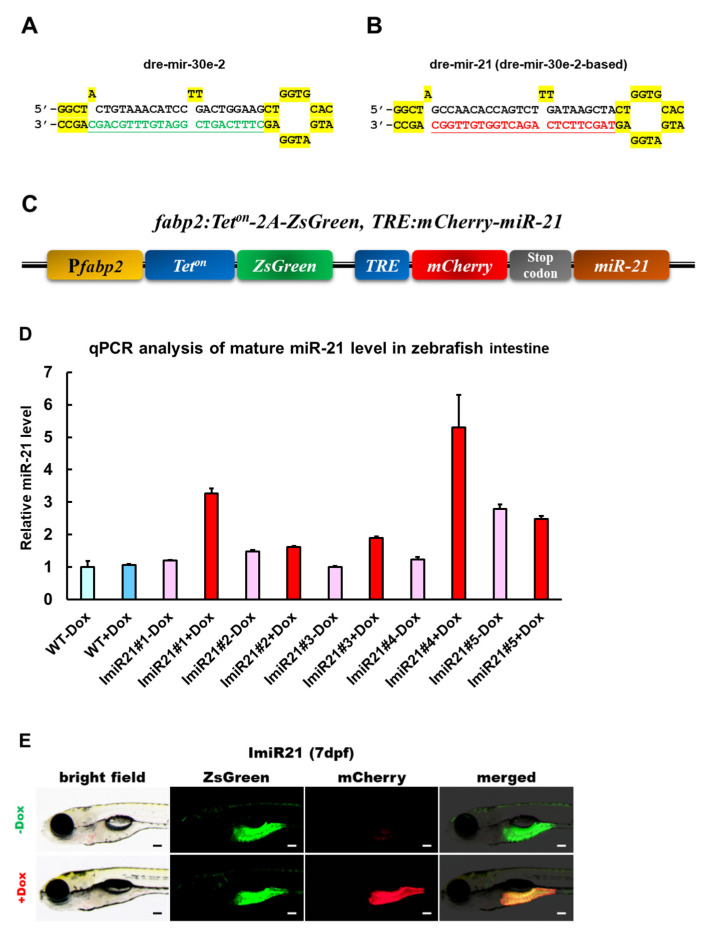
Generation of an intestine-specific and inducible microRNA (miR)-21 transgenic zebrafish. (**A**) Schematic diagram of the stem-loop structure of dre-mir-30e-2. The mature dre-miR-30e sequence (underlined) can be replaced by other mature miRNA sequences. (**B**) Schematic diagram of stem-loop structure of dre-mir-30e-2-based dre-mir-21. The mature miRNA region has been replaced by mature dre-mir-21. (**C**) Schematic diagram of the DNA construct used to generate ImiR-21 [*Tg*(*fabp2a:Teton-2A-ZsGreen, TRE:mCherry-miR-21*)]. (**D**) Relative quantification of miR-21 expression using RT-qPCR analysis. ImiR-21#1–5 + doxycycline (Dox) represents five independent transgenic lines. Control: wild-type (WT)—Dox and ImiR-21#1–5—Dox zebrafish. The expression level of miR-21 was normalized to that of *U6* in the intestine of the WT—Dox control. (**E**) Intestine-specific inducible miR-21 expression in ImiR-21#4 at 7 days post-fertilization (dpf). Transgenic larvae were treated with 25 μg/mL Dox from 3 to 7 dpf. Scale bar: 50 μm.

The stem-loop of dre-mir-30e-2 (Figure 1A) was modified to express miRNAs or siRNAs [43]. The mature miRNA sequence region in the stem-loop of dre-mir-30e-2 was replaced by the mature dre-mir-21 sequence (Figure 1B). The flanking sequences of dre-mir-30e-2 and dre-mir-30e-2-based dre-mir-21 stem-loops were synthesized in tandem by a gene synthesis service (Genomics CORP., Taiwan). The dre-mir-30e-2-based dre-mir-21 cassette was subcloned into the 3’ untranslated region (UTR) of the inducible and intestine-specific miRNA expression vector. The *fabp2a:Tet^on^-2A-ZsGreen, TRE:mCherry-miR-21* construct (Figure 1C) was used to establish the germline-transmitting transgenic zebrafish line ImiR-21 (*Tg (fabp2a:Tet^on^-2A-ZsGreen, TRE:mCherry-miR-21)*). The wild-type (WT) and ImiR-21 lines were treated with Dox (+Dox) at 3 dpf, and the expression level of intestinal miR-21 was analyzed using RT-qPCR at 7 dpf. The ImiR-21 lines harbor miR-21 genes that can be induced specifically in the intestine. The intestinal miR-21 expression in almost all ImiR-21 lines (ImiR-21#1–#4) without Dox treatment (−Dox) was similar to that in the WT (Figure 1D). The ImiR-21#4 + Dox larvae expressed a slightly higher level of miR-21 than other lines (#1–#3, and #5). ImiR-21#4 was used for subsequent experiments in this study. The transgenic line showed high ZsGreen signals without Dox treatment (−Dox), whereas both ZsGreen and mCherry signals were detected in the Dox treatment groups only (+Dox) (Figure 1E).

### 3.2. Identification of Intestinal Inflammation Markers in Zebrafish

**Figure 2:**
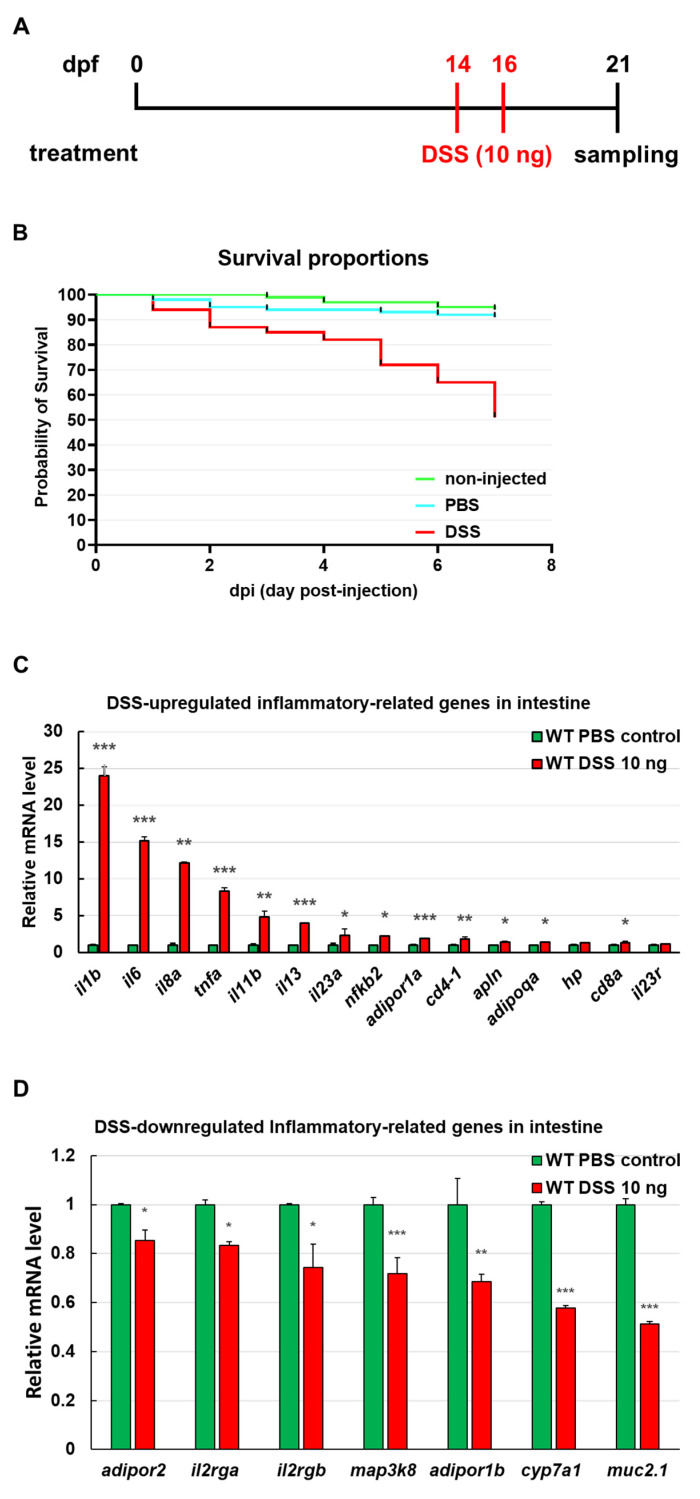
Identification of the gene markers of intestinal inflammation in zebrafish. (**A**) Schematic diagram of the dextran sodium sulfate (DSS) treatment strategy. Wild-type (WT) larvae were injected with PBS and 10 ng DSS twice at 14 and 16 days post-fertilization (dpf), and expression of the gene markers of intestinal inflammation was analyzed using RT-qPCR at 21 days post-fertilization (dpf). (**B**) Survival proportions of injected WT larvae from 0 dpi (days post-injection) to 7 dpi (14 to 21 dpf, *p* < 0.0001, *n* = 100 in three independent experiments). The survival rate of DSS-injected WT larvae was approximately 50%. The survival rates were not significantly different between non-injected and PBS-injected WT larvae (>90%). (**C**,**D**) RT-qPCR analysis of inflammation-related genes in the intestines of PBS- and DSS-injected WT lines at 21 dpf. mRNA expression levels were normalized to *gapdh* in the intestine of PBS-injected WT controls. Statistically significant differences from the PBS-injected WT control are denoted by * (*p* < 0.05), ** (*p* < 0.01), and *** (*p* < 0.001) for all panels.

miR-21 has been demonstrated to play a crucial role in the inflammatory pathway [26,44,45,46]. To determine the reactivity of inflammatory genes that are involved in CAC, WT larvae were injected with 10 ng DSS at 14 and 16 dpf to induce colitis (Figure 2A). The seven-day post-injection survival analysis showed significantly different survival rates (*p* < 0.0001, *n* = 100 in three independent experiments) of DSS-injected larvae (Figure 2B). Moreover, the expression levels of inflammatory genes were analyzed using RT-qPCR. The expression of some renowned inflammatory factors, such as *il1b*, *il6*, *il8a*, *tnfa*, *il11b*, *il13*, *il23a*, *nfkb1*, *adipor1a*, and *cd4-1* was significantly upregulated (Figure 2C). In addition, expression of anti-inflammatory genes, such as *muc2.1*, *cyp7a1*, *adipor1b*, *map3k8*, *il2rga*, *il2rgb*, and *adipor2* was repressed (Figure 2D).

### 3.3. Effects of Intestinal miR-21 Expression on Early Onset of IBD-Like Colitis

**Figure 3:**
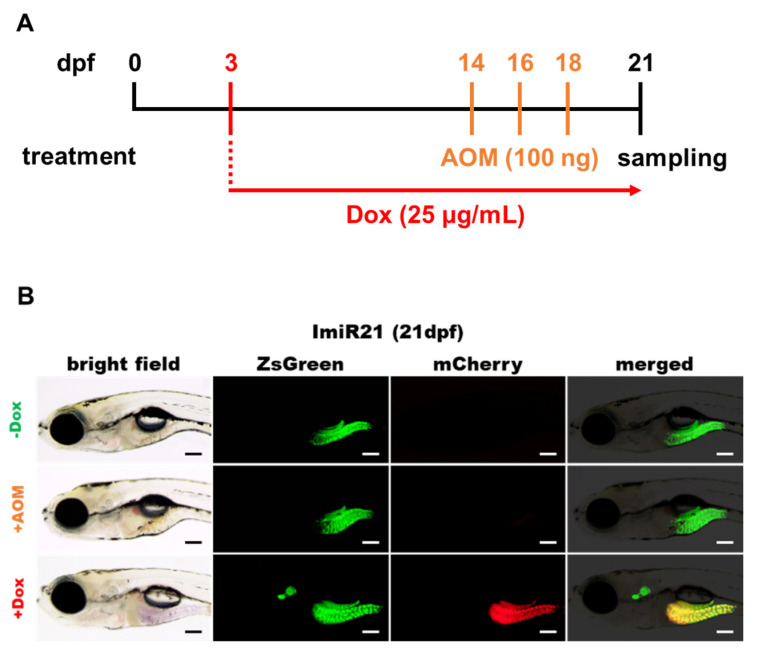

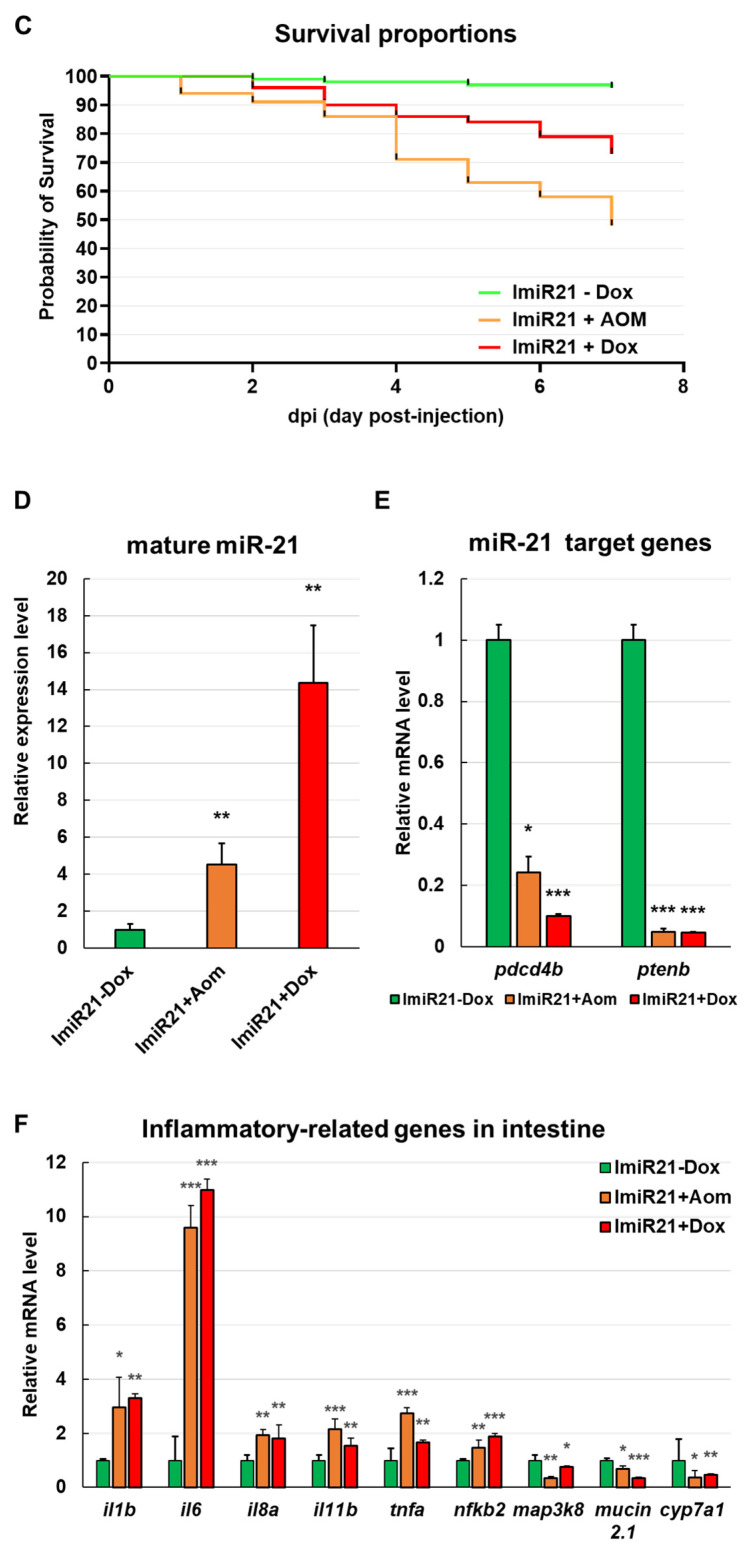
Characterization of expression profiles of inflammation-related genes in ImiR-21 larvae. (**A**) Schematic diagram of the strategy of doxycycline (Dox) and azoxymethane (AOM) treatment in ImiR-21. ImiR-21 + AOM: ImiR-21 larvae were injected with AOM (100 ng/4.6 nL) at 14, 16, and 18 days post-fertilization (dpf). ImiR-21 + Dox: ImiR-21 larvae were treated with 25 μg/mL Dox from 3 to 21 dpf. Morphology and gene regulatory patterns were analyzed at 21 dpf. (**B**) Intestinal morphology of ImiR-21 at 21 dpf. ImiR-21 expressed a strong ZsGreen signal in the zebrafish intestine. Upon Dox treatment, the mCherry (and miR-21) signal was induced in the intestine. The intestine was swollen in ImiR-21 + AOM and ImiR-21 + Dox. Scale bar: 200 μm. (**C**) Survival proportions of ImiR-21 from 14 to 21 dpf (*p* < 0.0001 *n* = 100 in 3 independent experiments). (**D**) RT-qPCR analysis of mature miR-21 level in intestine of ImiR-21 ± Dox and ImiR-21 + AOM at 21 dpf. *U6* was used as a reference gene for expression normalization. (**E**) The mRNA expression level of *pdcd4b* and *ptenb* in the intestines of ImiR-21 ± Dox and ImiR-21 + AOM at 21 dpf. (**F**) The mRNA expression level of inflammation-related genes in the intestine of ImiR-21 ± Dox and ImiR-21 + AOM at 21 dpf. Gene expression levels were normalized to *gapdh* levels in ImiR-21—Dox for panels (**E**,**F**). Statistically significant differences from PBS-injected WT control or ImiR-21—Dox are denoted by * (*p* < 0.05), ** (*p* < 0.01), and *** (*p* < 0.001) for panels (**D**–**F**).

DSS and AOM are extensively used as inflammation-related colon carcinogens in mouse models [47,48,49,50]. To investigate whether AOM could induce inflammation in the zebrafish intestine by upregulating the expression of miR-21, ImiR-21 larvae were injected with 100 ng AOM at 14, 16, and 18 dpf (Figure 3A). The intestine became swollen in AOM-injected and ImiR-21 + Dox (Figure 3B). The mortality rate of AOM-injected ImiR-21 was similar to that of DSS-injected WT zebrafish (Figure 3C and Figure 2B). In addition, the mortality rate also remarkably increased in Dox-induced (+Dox) overexpression of miR-21 in the early stage of ImiR-21. miR-21 levels in zebrafish intestines were analyzed by RT-qPCR in ImiR-21− Dox, ImiR-21 + AOM, and ImiR-21 + Dox at 21 dpf. miR-21 expression in the intestine was slightly upregulated by AOM compared to that in untreated ImiR-21 − Dox. Interestingly, the intestinal expression of miR-21 in ImiR-21 + Dox was much higher than that in AOM-treated larvae (Figure 3D). Our previous study indicated that *pdcd4b* and *ptenb* mRNAs are direct targets of miR-21 in zebrafish [42]. This result was reflected in the RT-qPCR data, which showed that increased levels of miR-21 repressed the expression of *pdcd4b* and *ptenb* mRNAs (Figure 3E). Furthermore, for the regulatory profiles of inflammatory genes in the intestine, ImiR-21 + Dox displayed similar features to DSS- or AOM-treated larvae (Figure 2C,D and Figure 3F).

### 3.4. Overexpression of miR-21 Causes Intestinal Epithelial Barrier Impairment

**Figure 4:**
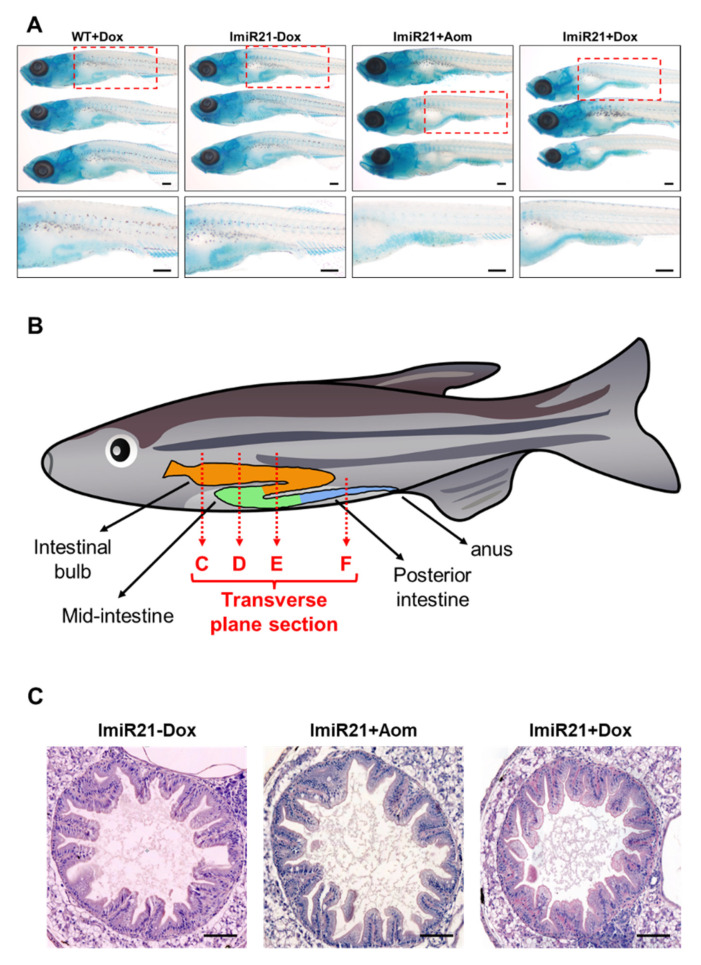

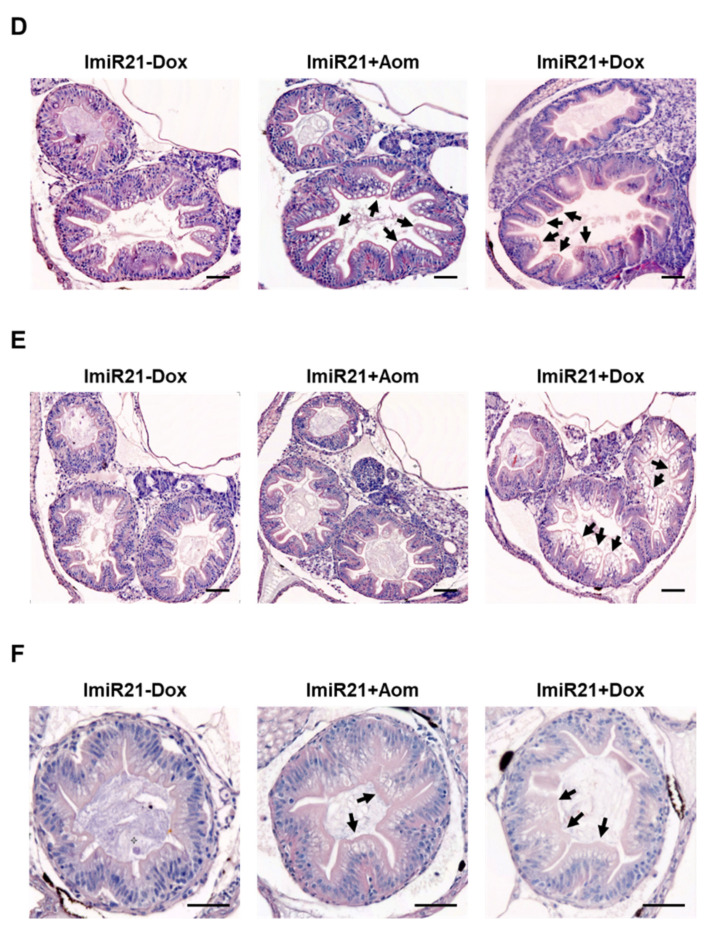
Characterization of inflammatory bowel disease (IBD)-like phenotype in ImiR-21. (**A**) Representative images of whole-mount alcian blue staining of WT + Dox, ImiR-21 ± Dox, and ImiR-21 + AOM at 21 days post-fertilization (dpf). Blue signals are the stained goblet cells in the injured villus of the posterior intestine of ImiR-21 + AOM and ImiR-21 + Dox animals under inflammatory conditions. Scale bar: 200 μm. (**B**) Schematic diagram of the intestinal structure of zebrafish. H&E-stained transverse plane sections in different parts of ImiR-21 ± Dox and ImiR-21 + AOM intestines (21 dpf) (intestinal bulb, mid-intestine, and posterior intestine are corresponded to panels (**C**–**F**)). Goblet cell hyperplasia is indicated by arrows. Scale bar: 50 μm.

Upregulation of miR-21 has been observed in various tissue inflammatory conditions [44,45] in human patients and animal models of IBD [26,46]. Overexpressed miR-21 leads to intestinal epithelial barrier impairment [51]; thus, we examined the early onset of intestinal inflammation in ImiR-21 fish. WT and ImiR-21 larvae were treated with Dox or AOM (Figure 3A). Whole-mount alcian blue staining displayed significant signals in the posterior intestine of ImiR-21 + AOM and ImiR-21 + Dox at 21 dpf (Figure 4A). Alcian blue stains acid mucins, which are secreted by various connective and epithelial tissue tumors. The presence of goblet cells in the injured villi indicates an inflammation in the posterior intestine of ImiR-21 + AOM and ImiR-21 + Dox. For histological analysis, paraffin-embedded zebrafish intestines at 21 dpf were sliced along the transverse plane into 4 μm sections (Figure 4B). Histopathological examination revealed goblet cell hyperplasia (GCH, indicated by arrows) in the villi of ImiR-21 + AOM and ImiR-21 + Dox (Figure 4C–F).

### 3.5. Chronic Effects of Intestinal miR-21 Expression on Colitis

**Figure 5:**
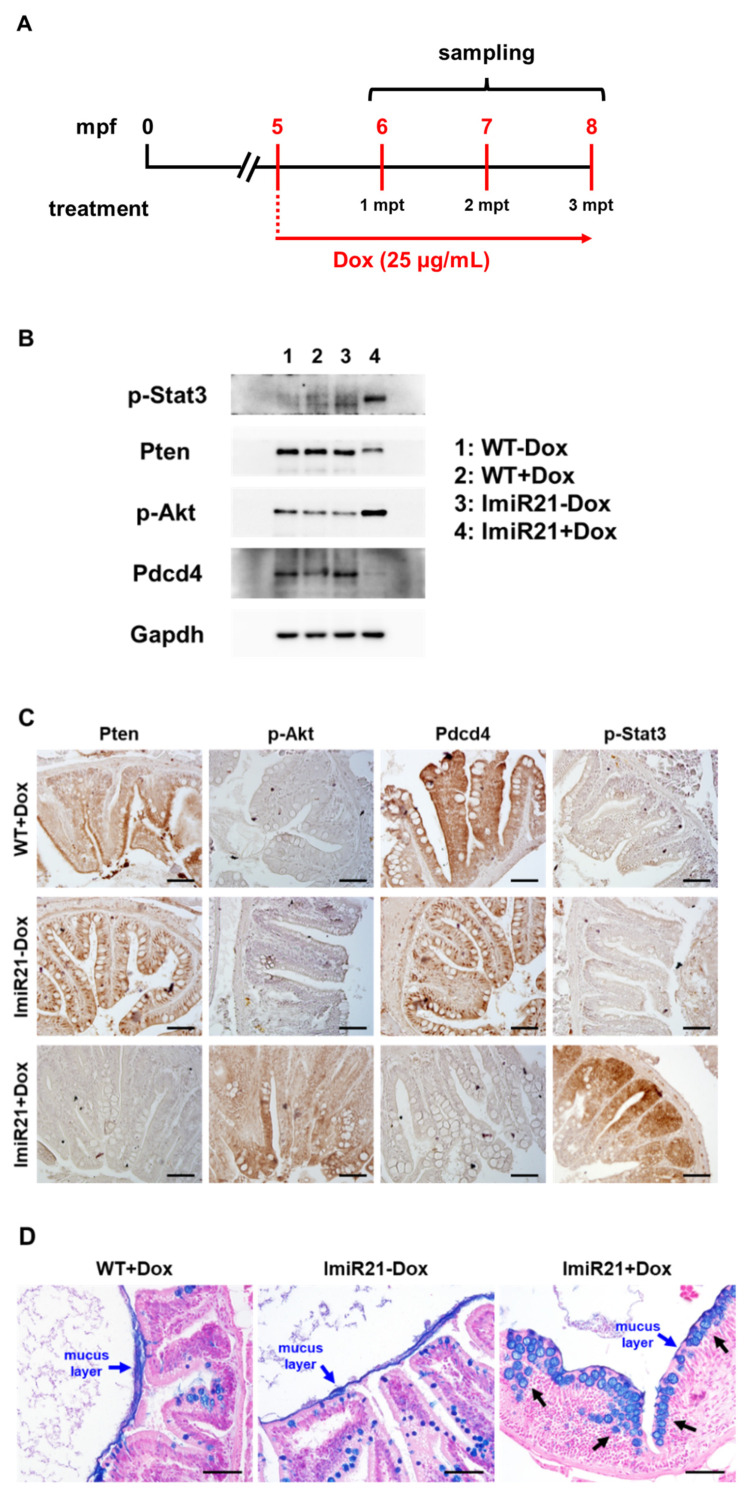
Chronic effects of intestinal miR-21 expression on colitis. (**A**) Schematic diagram of the Dox treatment strategy in adult ImiR-21. ImiR-21 larvae were treated with 25 μg/mL Dox from 5 to 8 months post-fertilization (mpf). (**B**) Representative Western blots of the intestinal protein of WT ± Dox and ImiR-21 ± Dox at 6 mpf/1 months post-treatment (mpt). (**C**) Expression patterns of Ptenb, p-Akt, Pdcd4, and p-Stat3 in 6 mpf/1 mpt WT + Dox and ImiR-21 ± Dox intestine samples after immunohistochemical staining. Scale bar: 50 μm. (**D**) Representative images of alcian blue-stained intestines of WT + Dox and ImiR-21 ± Dox at 6 mpf/1 mpt. Goblet cell hyperplasia (black arrow) and reduction of mucus layer (blue arrow) were observed in ImiR-21 + Dox intestine. Scale bar: 50 μm.

As ImiR-21 + Dox fish showed a similar gene regulatory feature to the intestinal carcinogen-treated fish, we analyzed whether the ImiR-21 intestine was predisposed to CAC development from the beginning. Because of the high mortality rate in the early stages of development of ImiR-21 with upregulated miR-21 expression, miR-21 was induced by Dox at 5 months post-fertilization (mpf) in the adult ImiR-21 intestine (Figure 5A). After one month of miR-21 induction, oncogenic p-Akt and p-Stat3 expression increased significantly, and the tumor-suppressive genes *pten* and *pdcd4* were substantially repressed in the intestine of ImiR-21 + Dox (Figure 5B,C and Figure S1). Alcian blue-stained sections indicate GCH (Figure 5D, black arrow) and reduction of the mucus layer (blue arrow) in ImiR-21 + Dox. These results indicated that the intestinal epithelial cells suffered from miR-21-mediated inflammation.

### 3.6. Chronic Effects of Intestinal miR-21 Expression on CAC

**Figure 6:**
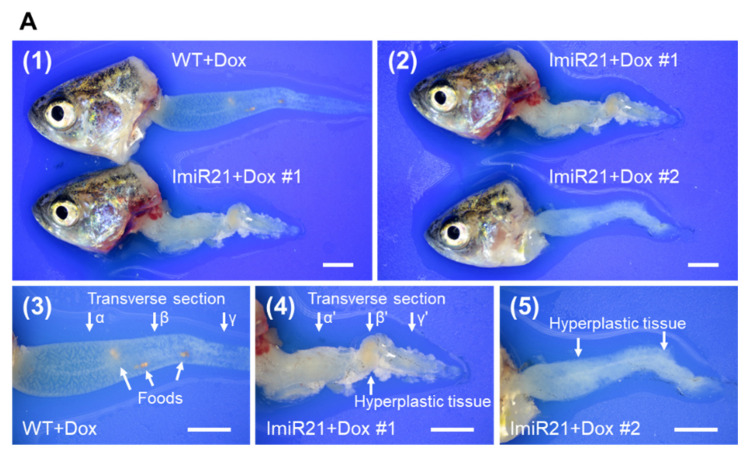

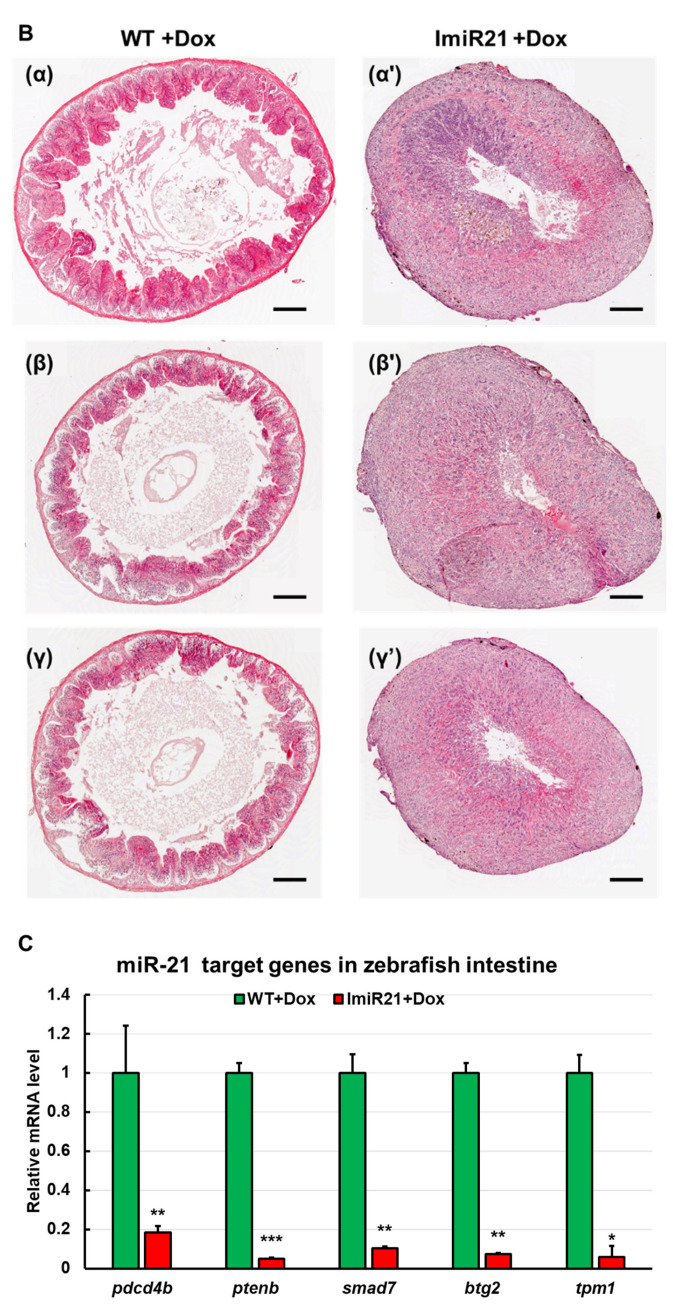

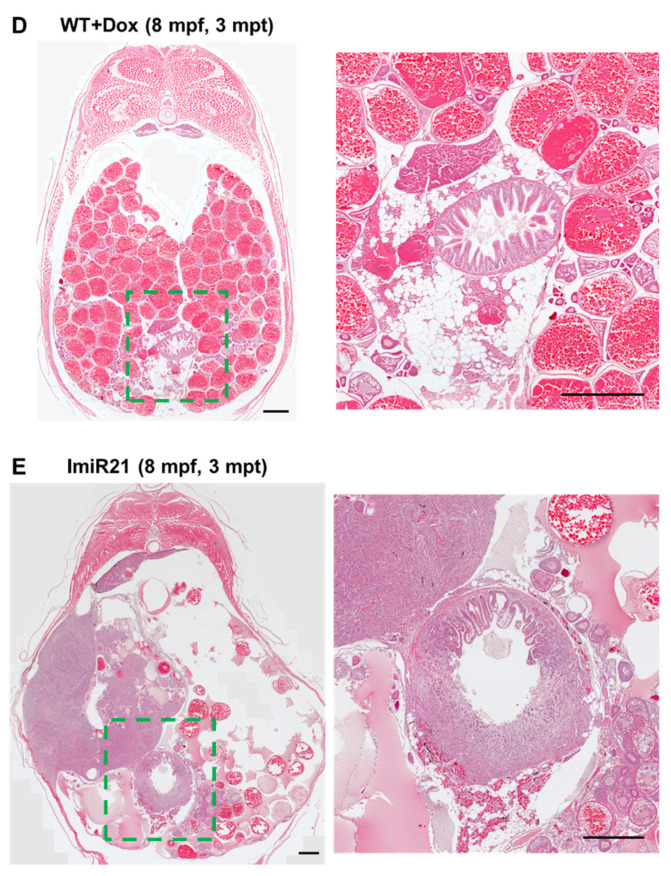
Chronic effects of intestinal miR-21 expression on colitis-associated cancer (CAC). (**A**) Representative images of gross intestinal phenotypes at 7 months post-fertilization (mpf)/2 months post-treatment (mpt) of WT + Dox and ImiR-21 + Dox. Scale bar: 2 mm. (**B**) H&E-stained intestinal sections of 7 mpf/2 mpt WT + Dox and ImiR-21 + Dox. (α and α’) Intestinal bulb. (β and β’) Mid-intestine. (γ and γ’) Edge of mid-intestine and posterior intestine. Scale bar: 250 μm. (**C**) RT-qPCR analysis of the mRNA expression of the CAC-associated genes in the intestine of 7 mpf/2 mpt WT + Dox and ImiR-21 + Dox. Gene expression levels were normalized to *gapdh* levels in WT + Dox. Statistically significant differences from WT + Dox are denoted by * (*p* < 0.05), ** (*p* < 0.01), and *** (*p* < 0.001). Representative images of H&E-stained sections of (**D**) WT + Dox and (**E**) ImiR-21 + Dox at 8 mpf/3 mpt. Scale bar: 2 mm.

ImiR-21 + Dox was weaker and more marasmic than the WT + Dox zebrafish (Figure S2A). The intestinal length of ImiR-21 + Dox was significantly shorter than that of WT + Dox at 2 months post-treatment (mpt)/7 mpf (Figure 6A panels 1 and 2 and Figure S2B). Bowels of WT + Dox were thin and translucent. There was no hyperplastic tissue in the intestines of WT + Dox (Figure 6A, panels 1 and 3). However, abundant hyperplastic tissues were observed in the intestines of ImiR-21 + Dox (Figure 6A, panels 1, 2, 4, and 5). Histological sections of Figure 6A panels 3 and 4 are shown in Figure 6B. Hyperplastic adenomas were observed in the intestine of ImiR-21 + Dox at 2 mpf (Figure 6B, panels α’, β’, and γ’). Furthermore, potential miR-21 target genes in CAC or CRC progression were all significantly repressed (Figure 6C). Long-term Dox treatment had no adverse effect on WT + Dox zebrafish (Figure 6D). However, a large invasive tumor was observed beside the posterior intestine of ImiR-21 + Dox at 3 mpt/8 mpf (Figure 6E).

### 3.7. miR21 Promotes CAC Development by Activating PI3K/AKT, IL-6/JAK/STAT3, and PDCD4/NF-κB/TNF-α (PSP) Signaling Networks

**Figure 7:**
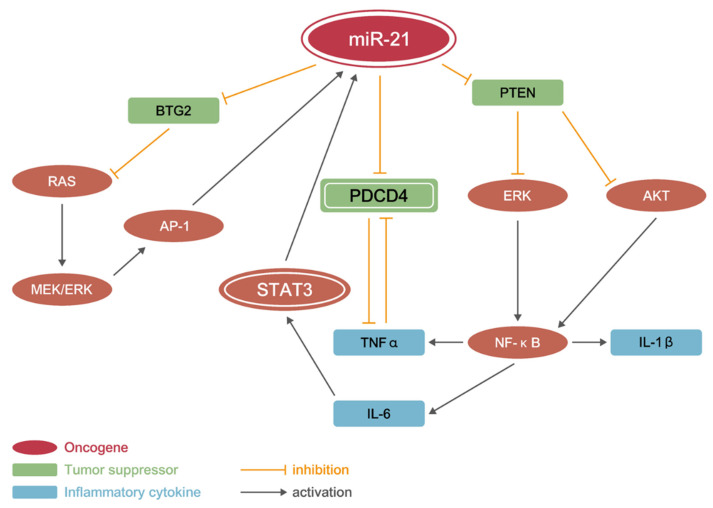
miR-21 Promotes CAC development by activating PI3K/AKT, STAT3, and PDCD4/TNF-α (PSP) signaling networks. Representative images of miR-21-mediated gene regulatory network in CAC/CRC development. miR-21 can induce CRC or colitis-associated cancer (CAC) in ImiR-21 through another PI3K/AKT, PDCD4/TNF-α, and IL-6/STAT3 signaling network, leading to initial inflammation. miR-21 and TNF-α repress PDCD4 and its tumor-suppressive activity; eventually, active STAT3 stimulates a strong inflammatory response and activates the invasion/metastasis process of tumor cells.

miR-21 is a well-known oncogenic miRNA that is upregulated in several human cancers. In this study, we compared the miR-21-mediated gene networks to DSS- or AOM-induced colitis in the zebrafish intestine and found that they have similar gene regulatory profiles during CAC development. By repressing Pten, miR-21 activates the Pi3k/Akt pathway and eventually the Nf-κB pathway. Activated Nf-κB promotes the release of the inflammatory cytokines Il-1β, Il-6, and Tnf-α. Il-6 activates Stat3 through the Jak signaling pathway; thus, p-Stat3 (active) binds to the promoter region of miR-21 and creates a positive feedback loop for miR-21. Tnf-α antagonizes Pdcd4. miR-21 targets tumor suppressor genes such as *pdcd4*, *btg2*, and *tpm1* to enhance cell proliferation, release more inflammatory cytokines, and activate oncogenes. Hence, the dysregulated gene networks and chronic inflammation mediated by miR-21 eventually lead to tumorigenesis during CAC development (Figure 7).

## 4. Discussion

IBD is a major risk factor for CAC and CRC [3,4,5,6,7,8]. In the traditional pathogenesis of GI neoplasia, epithelial cells or stem cells accumulate mutations in the *Wnt* signaling pathway regulators [52]. Loss of function of the tumor suppressor APC, which represses *Wnt* signaling, is the first step toward adenoma formation [53]. Interestingly, in some microorganism-induced tumors, TNF-α promotes nuclear accumulation of β-catenin without APC mutations [54]; these data provide a novel insight into the relationship between inflammation and tumorigenesis [55,56]. Similarly, proinflammatory signaling promotes β-catenin activation by activating Akt and NF-κB pathways [57,58].

miR-21 expression is upregulated in inflammatory conditions [26,44,45,46] and has been observed in inflammation-related diseases [26,27]. The gene regulatory profiles upon overexpression of miR-21 are strikingly similar to those observed in intestines of the intestinal carcinogen-treated lines ImiR-21 + AOM and ImiR-21 + Dox (Figure 3), suggesting that miR-21 leads to IBD-like symptoms in ImiR-21 + Dox larvae at 21 dpf (Figure 4). Previous studies have shown that the proinflammatory cytokines IL-6 and TNF-α [9,10,11,12,13,14,15,16,17,18,19] promote CAC tumorigenesis. In addition to the tumor suppressor APC, PDCD4 plays an inhibitory role in tumor growth, metastasis, and invasion [22]. Moreover, PDCD4 is associated with inflammatory diseases [23,24,25,59,60], and its presence may alleviate DSS-induced acute colitis in mice. The upregulated IL-6/STAT3 signaling pathway led to CAC in *Pdcd4*-knockout mice [20]; similar results were obtained in our transgenic zebrafish model (Figure 3 and Figure 5).

We demonstrated that miR-21 regulates at least three molecular and pathophysiological pathways during CAC development: Akt/Nf-κb, Pdcd4/Tnf-α, and Il-6/Stat3. First, miR-21 represses *pten* and activates Akt/Nf-κb pathways. Activated Aky/Nf-κb promotes the release of inflammatory cytokines, such as Il-6 and Tnf-α. Tnf-α not only promotes colon cancer cell migration and invasion [61] but also represses Pdcd4, and Pdcd4 deficiency may aggravate inflammatory response. Stat3 is activated by Il-6 through the Jak pathway. Activated Stat3 promotes the transcription of miR-21 and forms a positive feedback loop, which explains why miR-21 is upregulated in the AOM-treated intestine (Figure 3). Moreover, miR-21 directly targets several tumor suppressors, such as *pdcd4* and *btg2*, to enhance cell proliferation, release more inflammatory cytokines, and activate oncogenes. All the above events accelerate the early onset of tumorigenesis and metastasis in the ImiR-21 + Dox intestine (Figure 3 and Figure 5, Figure 6 and Figure 7). In our previous study, we showed that miR-21 targeted *smad7* to increase the phosphorylation of Smad3 in the zebrafish liver [42]. Another study showed that IL-6/JAK/STAT3 and TGF-β/SMAD pathways are required for the epithelial–mesenchymal transition in the early stages of cancer [62]. These studies further support our data wherein we observed a large invasive tumor in ImiR-21 + Dox within 3 months of miR-21 induction (Figure 6E).

The intestinal length of ImiR-21 + Dox was significantly shorter than that of the WT + Dox control at 2 mpt. Moreover, hyperplastic tissues and tumors were observed in the intestine of ImiR-21 + Dox (Figure 6 and Figure S2). Similar symptoms were observed in AOM/DSS-treated mouse models [63,64]. Hence, miR-21 has strong oncogenic effects and is critical for CAC and CRC development. Furthermore, since dysregulated miR-21 is easily detected in blood, serum miR-21 can be used as a promising biomarker for the early detection and prognosis of CRC [65,66], thereby providing an effective target for the development of novel therapeutic strategies to treat CRC.

## 5. Conclusions

In conclusion, our study emphasized the role of PSP signaling networks in inflammatory cytokine regulation, tumor suppressor modulation, and oncogenic activation. Our zebrafish model could successfully recreate the development of IBD to CAC/CRC with respect to physiological, metabolic, and histological aspects. Chronic inflammation is a critical risk factor for the development of tumors. Our findings, in addition to those of previous studies, contribute toward understanding the critical role of miR-21 in tumorigenesis. This knowledge would further help in developing novel therapeutics to target miR-21 to attenuate CRC/CAC progression.

## Data Availability

The data presented in this study are available in Supplementary Materials here.

## Supplementary Materials

The following are available online at https://www.mdpi.com/article/10.3390/cancers13215565/s1, Figure S1: Raw Western blots of (A) phosphorylated (p)-Stat3, (B) Ptenb, (C) p-Akt, (D) Pdcd4b, and (E) Gapdh in intestines of WT ± Dox and ImiR-21 ± Dox zebrafish at 6 mpf/1 mpt (months post-treatment); Figure S2: Dysregulated miR-21 leads to short bowel syndrome. (A) Representative images of morphology of WT + Dox and ImiR-21 + Dox at 7 mpf/2 mpt. ImiR-21 + Dox zebrafish were weaker and more marasmic than WT + Dox. (B) Representative images of intestinal length of WT + Dox and ImiR-21 + Dox at 7 mpf/2 mpt; Table S1: Primer sequences used for quantitative RT-PCR; Table S2 Number of zebrafish larvae for survival analysis.
