# Supplementary material for: MicroRNA-21 Plays Multiple Oncometabolic Roles in Colitis-Associated Carcinoma and Colorectal Cancer via the PI3K/AKT, STAT3, and PDCD4/TNF-α Signaling Pathways in Zebrafish
Source: Cancers (Basel). 2021 Nov 6;13(21):5565. doi: 10.3390/cancers13215565 (PMC8583575; doi:10.3390/cancers13215565)
Supplement: Supplementary file 1 [file cancers-13-05565-s001.zip › 20211105_cancers-1446062-supplementary.pdf]

cancers

MDPI

Supplementary Materials: MicroRNA-21 Plays Multiple Oncometabolic Roles in Coli-tis-Associated Carcinoma and Colorectal Cancer via the PI3K/AKT, STAT3, and PDCD4/TNF-α Signaling Pathways

Chi-Yu Lai, Kun-Yun Yeh, Bi-Feng Liu, Tzu-Ming Chang, Chuan-Hsun Chang, Yung-Feng Liao, Yi-Wen Liu and Guor Mour Her.

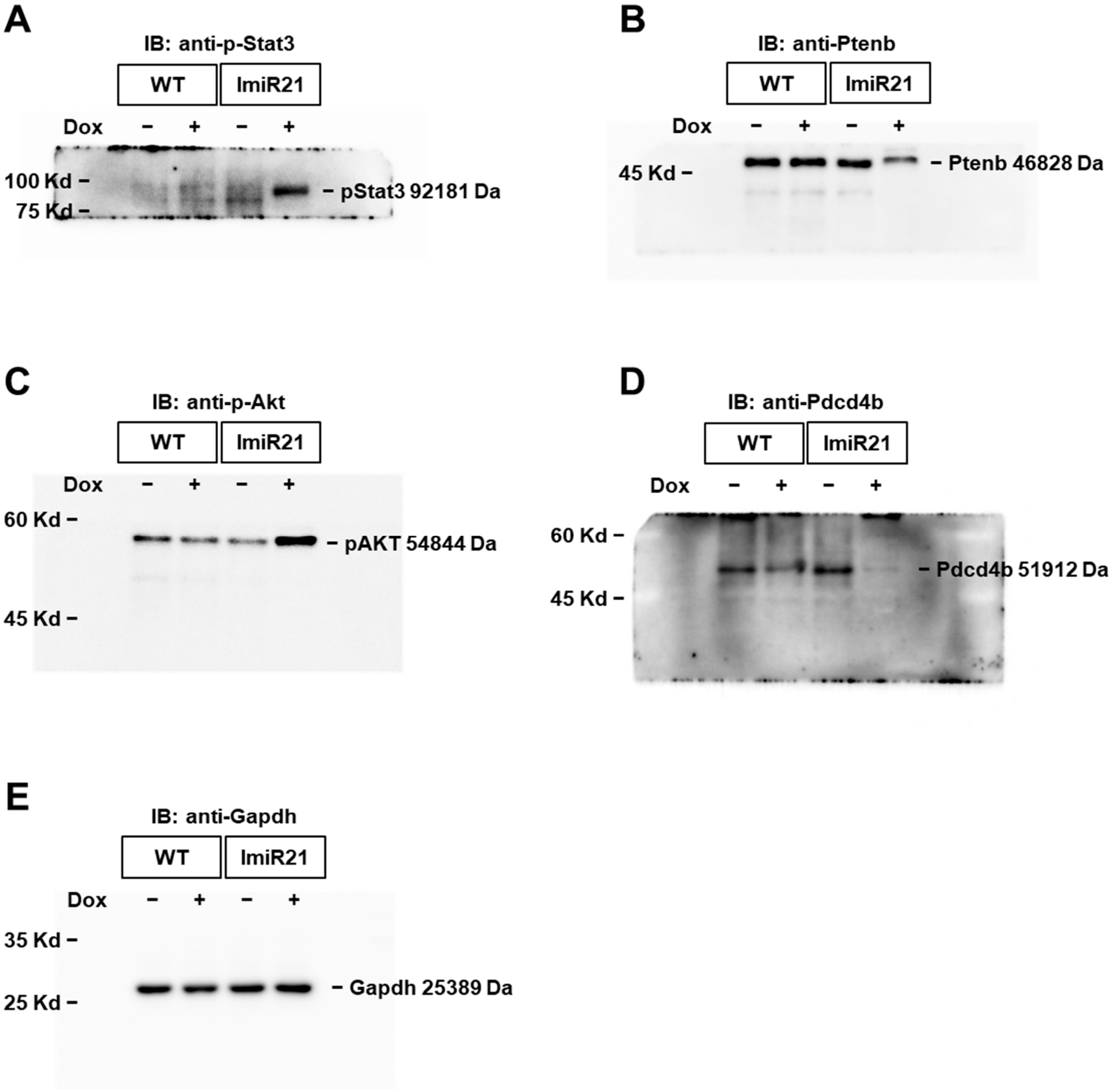

Figure S1. Raw western blots of (**A**) phosphorylated (p)-Stat3, (**B**) Ptenb, (**C**) p-Akt, (**D**) Pdcd4b, and (**E**) Gapdh in intestines of WT ± Dox and ImiR-21 ± Dox zebrafish at 6 mpf/1 mpt (months post-treatment).

A

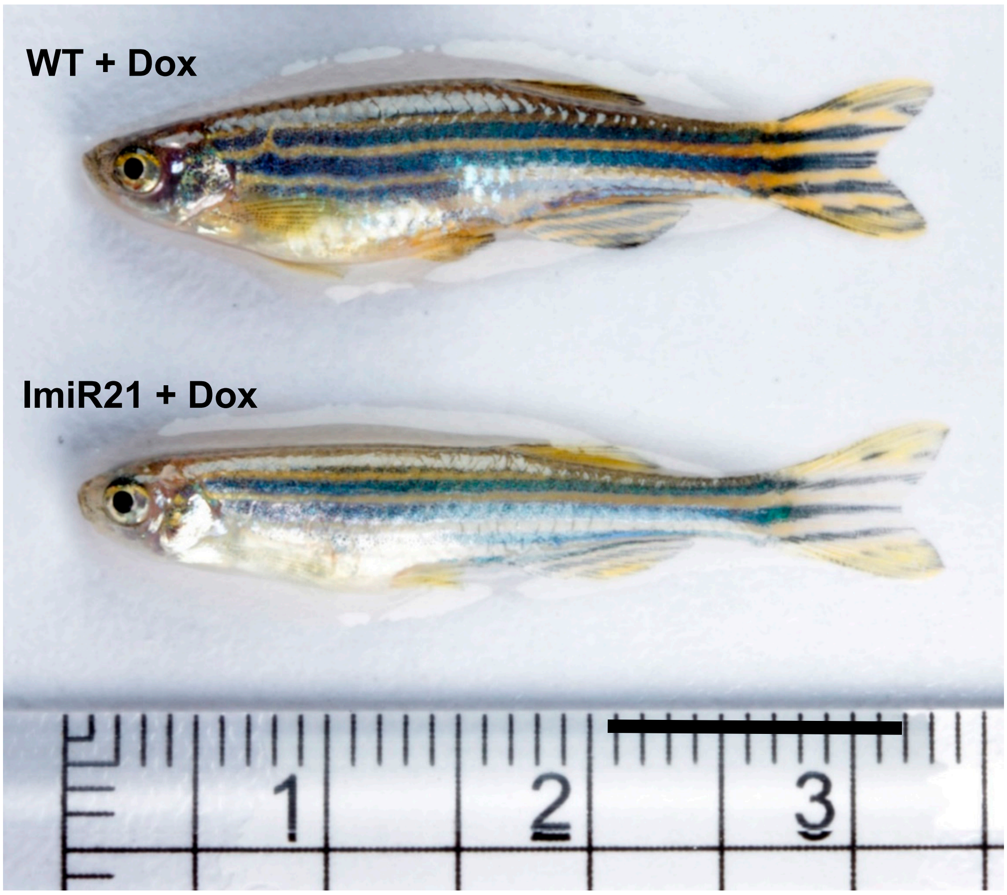

B

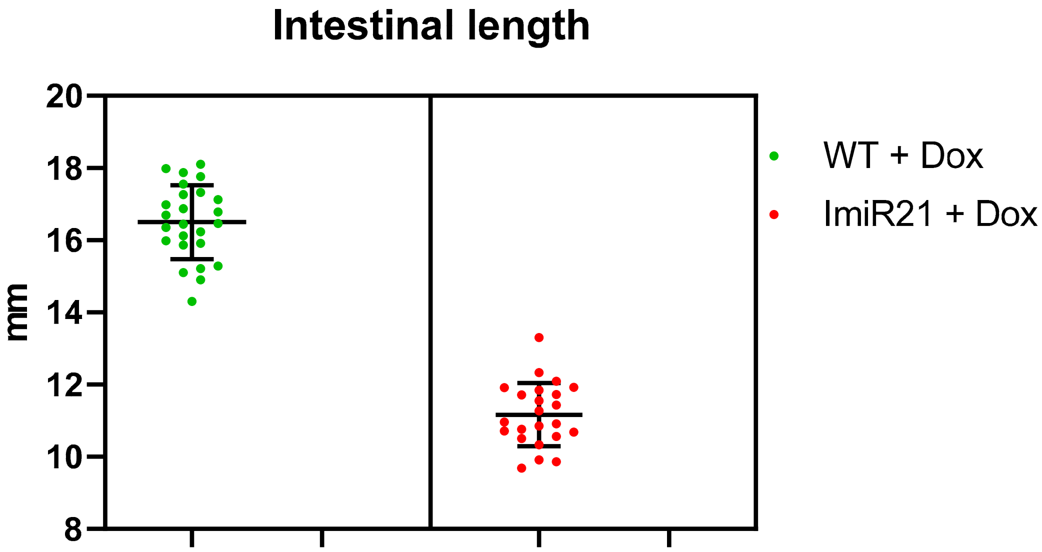

Figure S2. Dysregulated miR-21 leads to short bowel syndrome. (**A**) Representative images of morphology of WT + Dox and ImiR-21 + Dox at 7 mpf/2 mpt. ImiR-21 + Dox zebrafish were weaker and more marasmic than WT + Dox. (**B**) Representative images of intestinal length of WT + Dox and ImiR-21 + Dox at 7 mpf/2 mpt.

Table S1. Primer sequences used for quantitative RT-PCR.

Gene.	Accession	Forward Primer	Reverse Primer
gapdh	NM_001115114.1	GTGGAGTCTACTGGTGTCTTC	GTGCAGGAGGCATTGCTTACA
U6		TTGGTCTGATCTGGCACATATAC	AAAAATATGGAGCGCTTCACG
miR-21-RT	NR_030020.1 and NR_030021.2	GAAAGAAGGCGAGGAGCAGATCGAGGAAGAAGACGGAA-GAATGTGCGTCTCGCCTTCTTTCGCCAACAC	
miR-21		GCAGCTAGCTTATCAGACTGGTGT	CGAGGAAGAAGACGGAAGAAT
adipoqa	XM_009297486	CTGAAAGGAGAAAGTGGAGAGG	GTAGAAGGTTTGGTGAAGCG
adipor1a	XM_005169284	GGAGAAGATGGAGGAGTTTGTG	AAACAGGCACGGAAGGAG
adipoR1-A	NM_0010024678	TGTGCATAAGGTTTGGGAGG	AGGTAGTCGTTGTCTTTTAGCC
adipoR1-B	BC044388	GTCTGTCGTATGTGTTCTGGG	GGTAAAGTGCATTGTGGGAAC
adipoR2	BC154262	CGCCATCACAGTATCCCAG	CAATCATAAAGTGCAGCGTGG
adipor2	NM_001025506	TCTCTGTTCTCTATTTTGCCCC	CCATCAAACAGCAATAGACCAAG
apelin	NM_001166124	TCTTTGGGCACCGTGAATAC	CGTTCTGACCTACTGATCCATG
btg2	NM_130922.2	CGCAGCTCCAGGTGTTCA	AATGGTGCTGGTAGTGTTCTGAGA
cd4-1	NM_001135096.1	GTCACCCTACCCAGAGAAAAG	TGCGTTTGTCCCCTTACTG
cd8a	NM_001040049.1	TCGGAGGTTGTGGACTTTTC	GTGGGCATTTTCGCATGAC
cyp7a1	NM_201173	GATCCATTCTCCTACCATGCTG	GGTGTAACCTTGACTGGGATC
hp	XM_689364.3	ACGGAAGTAGCAGTGGAAAAG	GGAAGTGGGATGGGCAATATAG
il-11b	XM_021468285	ACGGAATAGTGACGAATTGGG	CAATGTGGGATGGACGATCTC
il-11b	NM_212844	TGGCGAACGTCATCCAAG	GGAGCACTGGGCGACGCATA
il-13	NM_001199905	CCCAAAAGAGACAAAGGCAAAAG	CAAATTTGACACCGGACAGG
il-1b	AY340959.1	TCAAACCCCAATCCACAGAG	TCACTTCACGCTCTTGGATG
il-1b	XM_021468285	ACGGAATAGTGACGAATTGGG	CAATGTGGGATGGACGATCTC
il-23a	FN869917	ATGATCAGGAATCGGCAGTG	GAGAAGCCAGGTATGAGAACAC
il-23r	NM_001113506	CAATGGCATTTCACACCCTG	TTCTTGGCTCAGTTCATCCC
il-2rga	NM_001128271	GCTGAATTGTGGCTTTACTGG	AAACTGTATGTGGTCCTGACG
il-2rgb	NM_001123050	GAGCCGTCCTGTGATGTATG	AGATTTCGGTTTGAGGACAGG
il-6	NM_001261449.1	CAGAGACGAGCAGTTTGAGAG	AGACATCTTTCCGTGCTGAAG
il-8a	XM_009306855.3	CATTGAAACAGAAAGCCGACG	GATCTCCTGTCCAGTTGTCATC
map3k8	XM_687085	AGGACACCAAAACTCGGAAG	CACAGCAAGGCCCCATATAG
muc2.1	XM_021470771	CCAAGATGAAGGACAAGGAGAG	GGAGAAAACCGTACACAGACA
nf-κb2	NM_001001840.3	ATGACGAGCCTCTTTTCCTG	TCTCTTCCGTGGGTTAAACAC
pdcd4b	NM_198978.1	TCAGATGAGACGGGGCTTTG	CTTACGGCCCCGACTAGGAC
smad7	NM_175082.3	ACCAGACAGTTCATCAGCAG	ATCCTCTGGAGAACAAACGG
tnfa	NM_212859.2	GGTGTTTGGGATCATTTTGGC	GCCTTGGAAGTGAAATTGCC
tpm1	XM_005174375.4	TGAGCAGGCTGAGGGAGA	CGACGCTCTGGTACTCCT

Table S2. Number of zebrafish larvae for survival analysis.

n = 100, 3 independent experiments for every analysis					
**WT-non injected**	**I**	**II**	**III**	**Average**	
**dpf / dpi**	**Death**	**Death**	**Death**	**Raw**	**Round**
**14 / 0**	0	0	0	0.00	0
**15 / 1**	1	0	0	0.33	0
**16 / 2**	0	0	1	0.33	0
**17 / 3**	1	1	0	0.67	1
**18 / 4**	2	1	2	1.67	2
**19 / 5**	0	0	1	0.33	0
**20 / 6**	1	2	2	1.67	2
**21 / 7**	1	1	1	1.00	1
**WT-PBS**	**I**	**II**	**III**	**Average**	
**dpf / dpi**	**Death**	**Death**	**Death**	**Raw**	**Round**
**14 / 0**	0	0	0	0.00	0
**15 / 1**	2	1	2	1.67	2
**16 / 2**	3	3	3	3.00	3
**17 / 3**	2	1	1	1.33	1
**18 / 4**	1	0	0	0.33	0
**19 / 5**	1	1	0	0.67	1

**20 / 6**	1	0	1	0.67	1
**21 / 7**	1	1	0	0.67	1
**WT-DSS**	**I**	**II**	**III**	**Average**	
**dpf / dpi**	**Death**	**Death**	**Death**	**Raw**	**Round**
**14 / 0**	0	0	0	0.00	0
**15 / 1**	9	5	5	6.33	6
**16 / 2**	9	6	5	6.67	7
**17 / 3**	3	2	2	2.33	2
**18 / 4**	3	4	2	3.00	3
**19 / 5**	10	11	8	9.67	10
**20 / 6**	7	9	6	7.33	7
**21 / 7**	12	17	13	14.00	14
**ImiR21 - Dox**	**I**	**II**	**III**	**Average**	
**dpf / dpi**	**Death**	**Death**	**Death**	**Raw**	**Round**
**14 / 0**	0	0	0	0.00	0
**15 / 1**	0	1	0	0.33	0
**16 / 2**	1	1	0	0.67	1
**17 / 3**	0	2	1	1.00	1
**18 / 4**	0	1	0	0.33	0
**19 / 5**	1	1	1	1.00	1
**20 / 6**	0	0	1	0.33	0
**21 / 7**	0	2	1	1.00	1
**ImiR21 + AOM**	**I**	**II**	**III**	**Average**	
**dpf / dpi**	**Death**	**Death**	**Death**	**Raw**	**Round**
**14 / 0**	0	0	0	0.00	0
**15 / 1**	6	7	6	6.33	6
**16 / 2**	2	3	3	2.67	3
**17 / 3**	4	6	5	5.00	5
**18 / 4**	14	17	15	15.33	15
**19 / 5**	8	9	8	8.33	8
**20 / 6**	4	6	5	5.00	5
**21 / 7**	9	12	10	10.33	10
**ImiR21 + Dox**	**I**	**II**	**III**	**Average**	
**dpf / dpi**	**Death**	**Death**	**Death**	**Raw**	**Round**
**14 / 0**	0	0	0	0.00	0
**15 / 1**	0	1	0	0.33	0
**16 / 2**	3	6	4	4.33	4
**17 / 3**	6	7	7	6.67	7
**18 / 4**	5	4	4	4.33	4
**19 / 5**	2	2	3	2.33	2
**20 / 6**	5	4	7	5.33	5
**21 / 7**	6	6	7	6.33	6
