# Supplementary material for: MicroRNA-21 Plays Multiple Oncometabolic Roles in Colitis-Associated Carcinoma and Colorectal Cancer via the PI3K/AKT, STAT3, and PDCD4/TNF-α Signaling Pathways in Zebrafish
Source: Cancers (Basel). 2021 Nov 6;13(21):5565. doi: 10.3390/cancers13215565 (PMC8583575; doi:10.3390/cancers13215565)
Supplement: Supplementary file 1 [file cancers-13-05565-s001.zip › cancers-1446062 Original Images for Blots/Original Images for Blots/Original Images for Blots.pptx]

## Slide 1
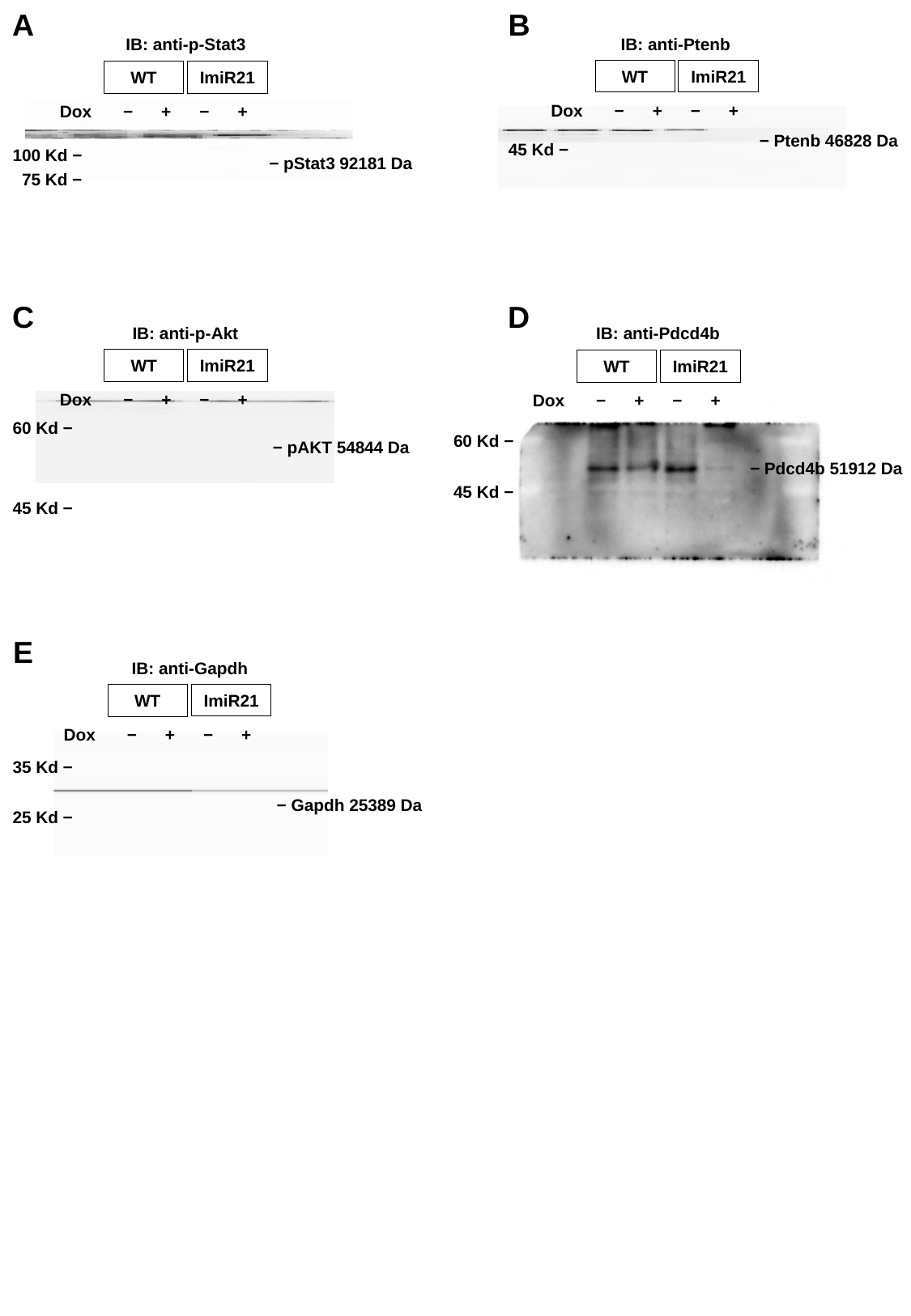

A
B
IB: anti-Ptenb
ImiR21
WT
Dox
−
+
−
+
− Ptenb 46828 Da
45 Kd −
IB: anti-p-Stat3
ImiR21
WT
Dox
−
+
−
+
100 Kd −
− pStat3 92181 Da
75 Kd −
C
IB: anti-p-Akt
ImiR21
WT
Dox
−
+
−
+
60 Kd −
− pAKT 54844 Da
45 Kd −
D
IB: anti-Pdcd4b
ImiR21
WT
Dox
−
+
−
+
60 Kd −
− Pdcd4b 51912 Da
45 Kd −
E
IB: anti-Gapdh
ImiR21
WT
Dox
−
+
−
+
35 Kd −
− Gapdh 25389 Da
25 Kd −
