# Supplementary figures and images for: MicroRNA-21 Plays Multiple Oncometabolic Roles in Colitis-Associated Carcinoma and Colorectal Cancer via the PI3K/AKT, STAT3, and PDCD4/TNF-α Signaling Pathways in Zebrafish
Source: Cancers (Basel). 2021 Nov 6;13(21):5565. doi: 10.3390/cancers13215565 (PMC8583575; doi:10.3390/cancers13215565)

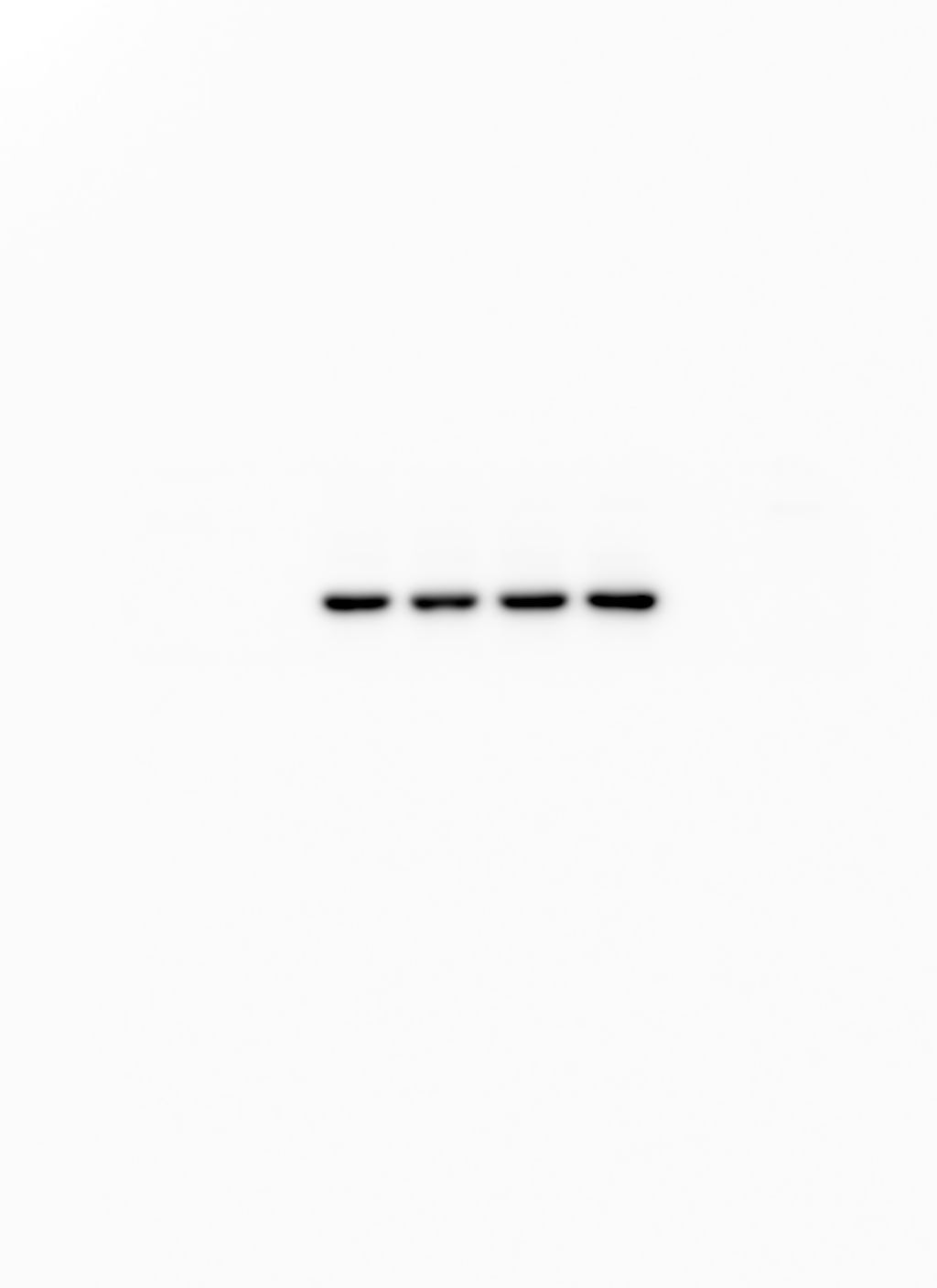

Supplement: Supplementary file 1 [file cancers-13-05565-s001.zip › cancers-1446062 Original Images for Blots/Original Images for Blots/GAPDH.tif]

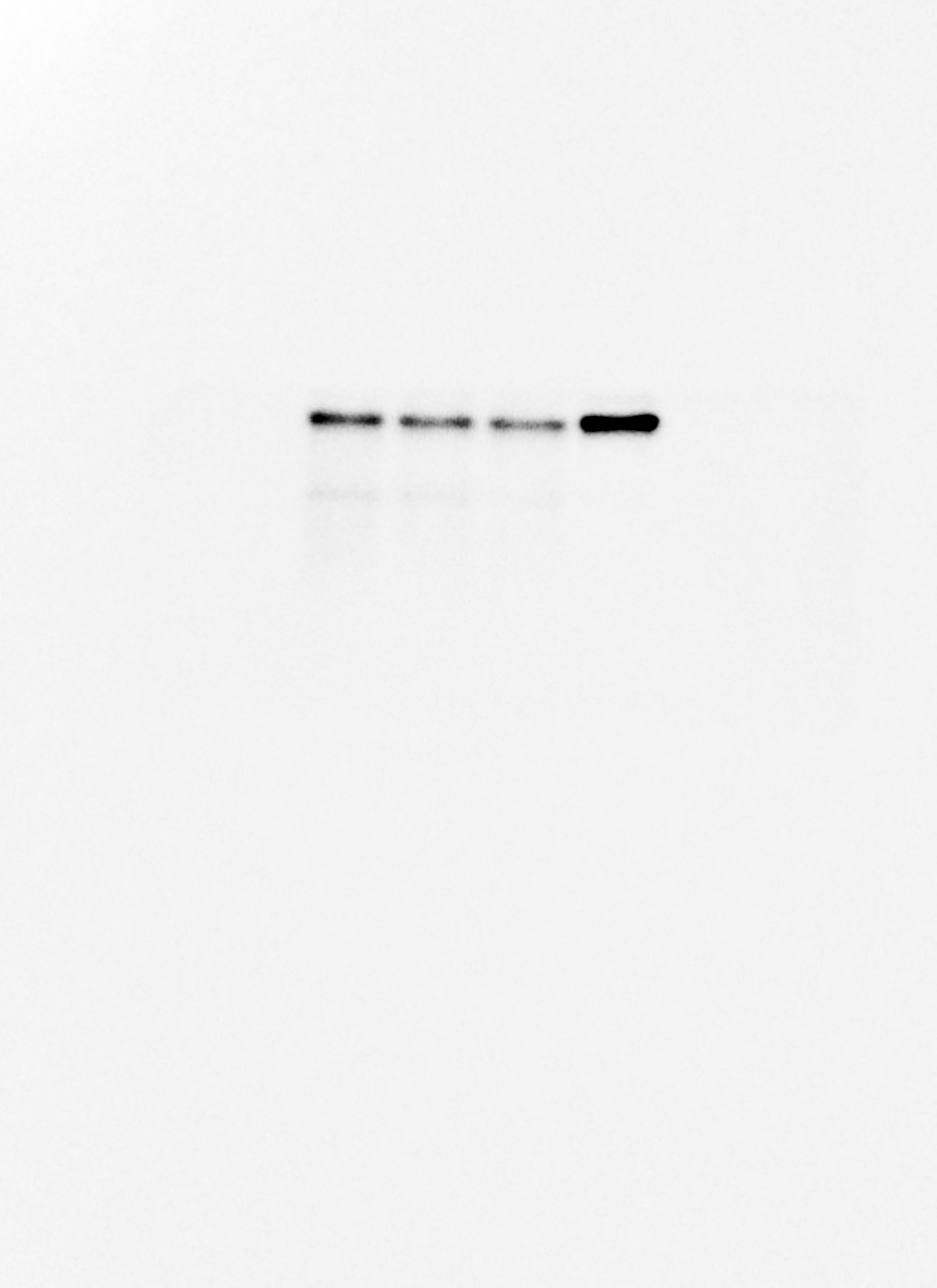

Supplement: Supplementary file 1 [file cancers-13-05565-s001.zip › cancers-1446062 Original Images for Blots/Original Images for Blots/pAKT.tif]

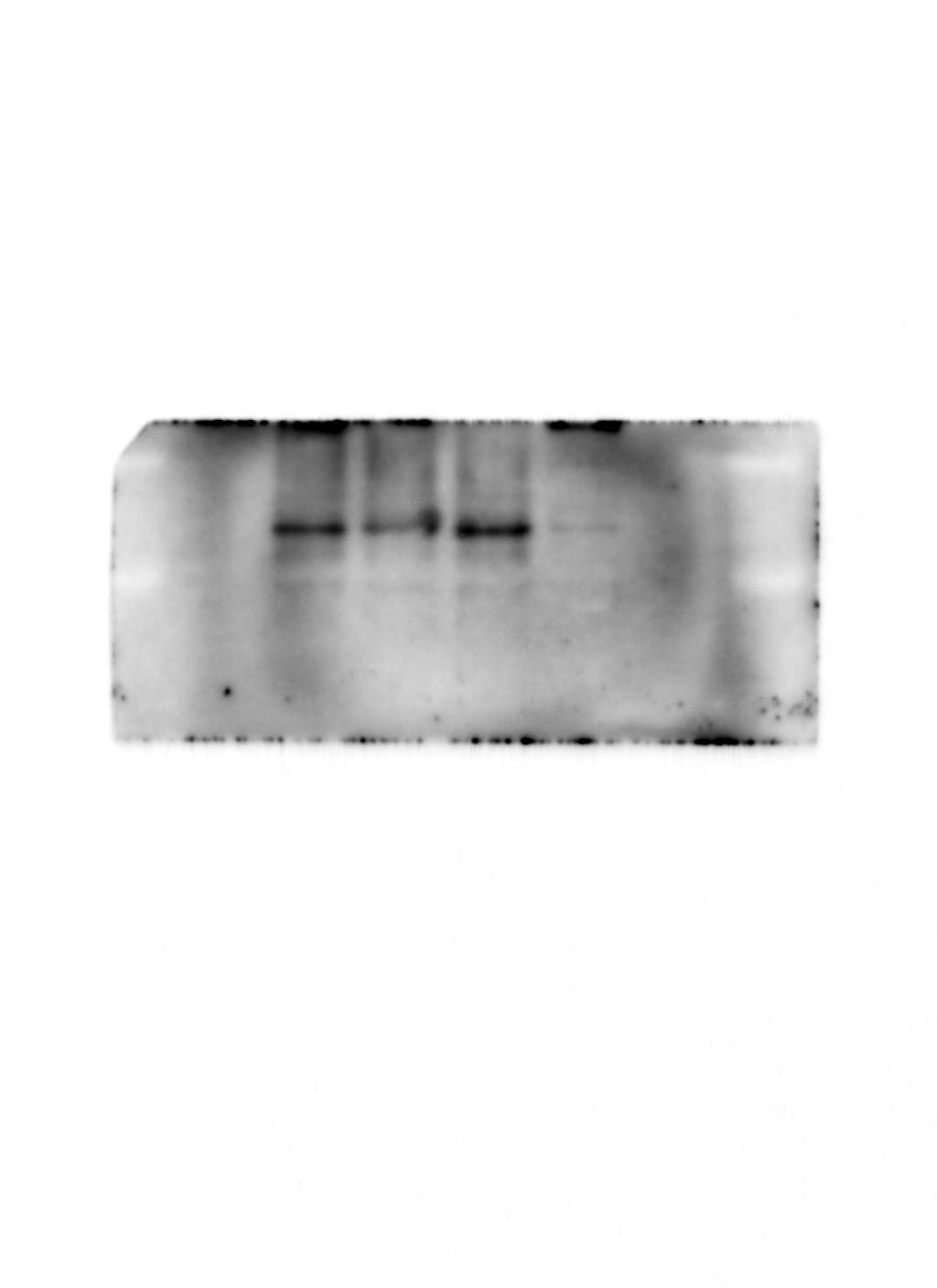

Supplement: Supplementary file 1 [file cancers-13-05565-s001.zip › cancers-1446062 Original Images for Blots/Original Images for Blots/PDCD4.jpg]

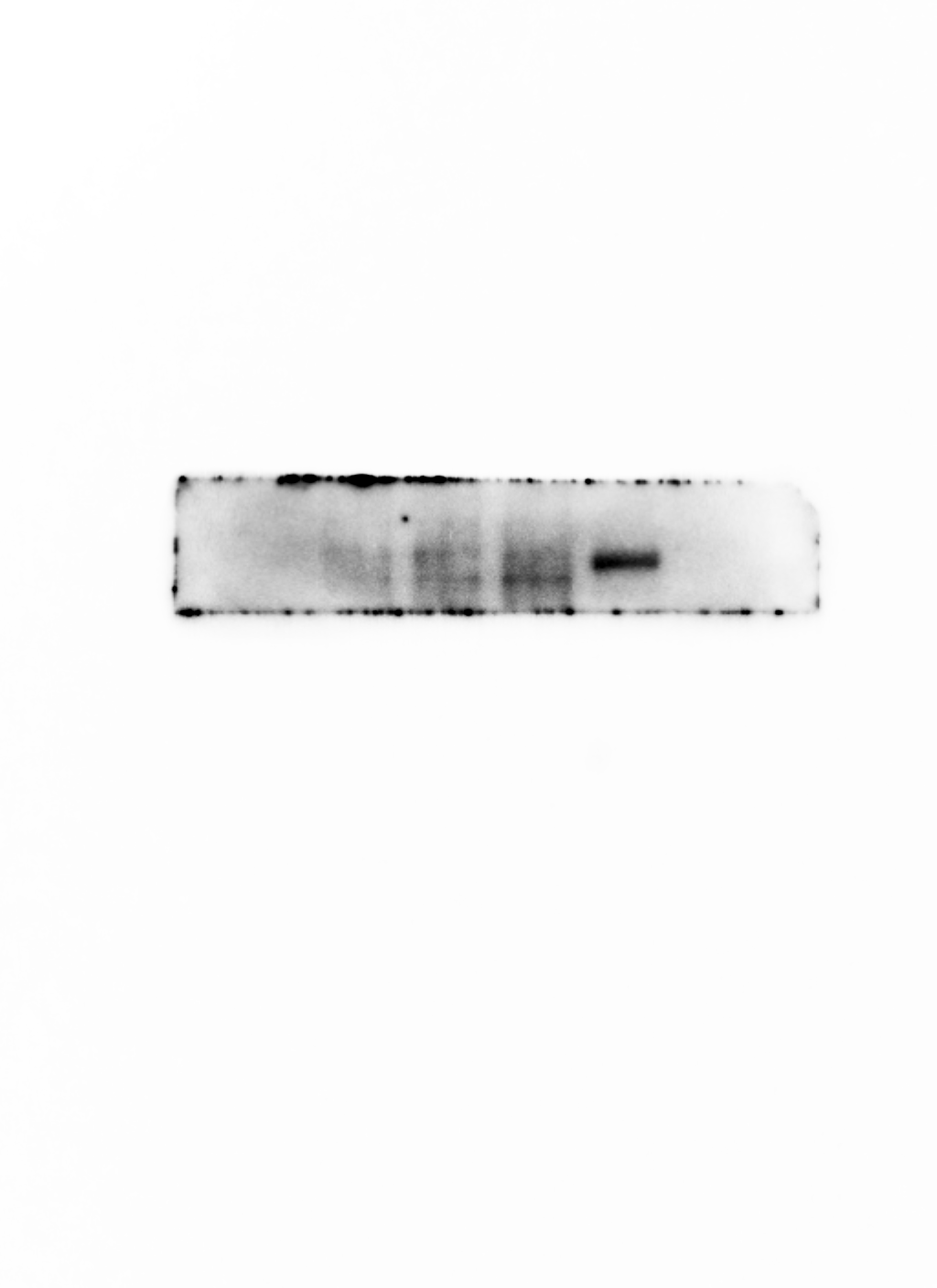

Supplement: Supplementary file 1 [file cancers-13-05565-s001.zip › cancers-1446062 Original Images for Blots/Original Images for Blots/pSTAT3.tif]

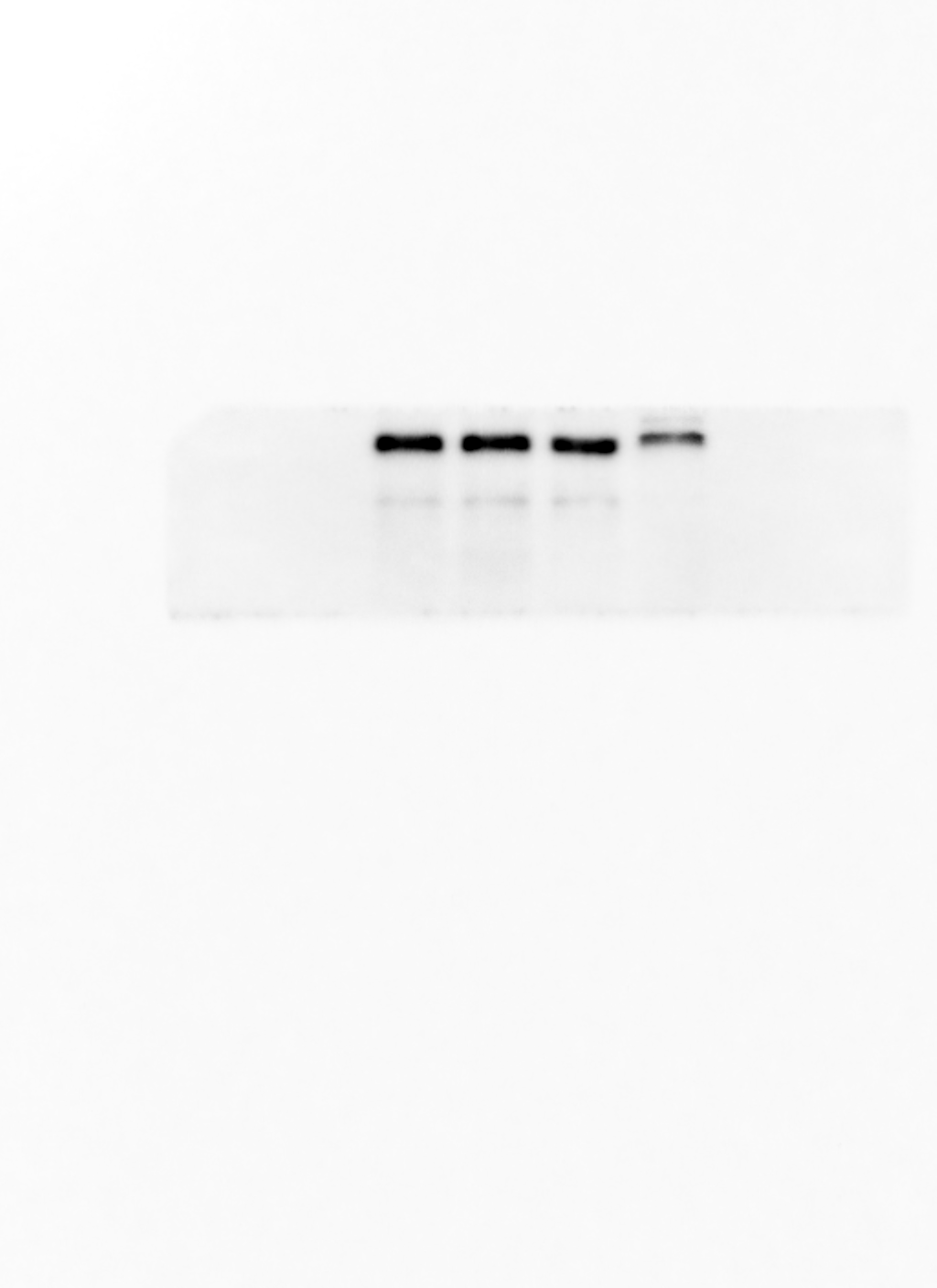

Supplement: Supplementary file 1 [file cancers-13-05565-s001.zip › cancers-1446062 Original Images for Blots/Original Images for Blots/PTEN.tif]
